# Virtual Reality as a Tool for Evaluation of Repetitive Rhythmic Movements in the Elderly and Parkinson's Disease Patients

**DOI:** 10.1371/journal.pone.0030021

**Published:** 2012-01-18

**Authors:** Pablo Arias, Verónica Robles-García, Gabriel Sanmartín, Julian Flores, Javier Cudeiro

**Affiliations:** 1 Neuroscience and Motor Control Group (NEUROcom), Department of Medicine-INEF Galicia, University of A Coruña, A Coruña, Spain; 2 Instituto de Investigacións Tecnolóxicas, University of Santiago de Compostela, Santiago de Compostela, Spain; Mayo Clinic, United States of America

## Abstract

This work presents an immersive Virtual Reality (VR) system to evaluate, and potentially treat, the alterations in rhythmic hand movements seen in Parkinson's disease (PD) and the elderly (EC), by comparison with healthy young controls (YC). The system integrates the subjects into a VR environment by means of a Head Mounted Display, such that subjects perceive themselves in a virtual world consisting of a table within a room. In this experiment, subjects are presented in 1^st^ person perspective, so that the avatar reproduces finger tapping movements performed by the subjects. The task, known as the finger tapping test (FT), was performed by all three subject groups, PD, EC and YC. FT was carried out by each subject on two different days (sessions), one week apart. In each FT session all subjects performed FT in the real world (FT_REAL_) and in the VR (FT_VR_); each mode was repeated three times in randomized order. During FT both the tapping frequency and the coefficient of variation of inter-tap interval were registered. FT_VR_ was a valid test to detect differences in rhythm formation between the three groups. Intra-class correlation coefficients (ICC) and mean difference between days for FT_VR_ (for each group) showed reliable results. Finally, the analysis of ICC and mean difference between FT_VR_ vs FT_REAL_, for each variable and group, also showed high reliability. This shows that FT evaluation in VR environments is valid as real world alternative, as VR evaluation did not distort movement execution and detects alteration in rhythm formation. These results support the use of VR as a promising tool to study alterations and the control of movement in different subject groups in unusual environments, such as during fMRI or other imaging studies.

## Introduction

Virtual Reality (VR) is a technique by which a person interacts with an artificial reality, which can be totally controlled by the experimenter, so that responses of the subject can be perfectly monitored and evaluated [1]. VR technology emulates the real world in an environment where characteristics are controlled, measurable and modifiable [2], permitting the isolation of subjects from non-natural research environments like fMRI scans, or complex laboratory settings [3]–[5].

This is acquired by means of creating a virtual environment (VE), so that the experimental subject is integrated into the VE by providing virtual information through appropriate sensory channels; eyes (head-mounted-displays (HMD); skin (haptic devices) [6]; or hearing (aural devices). This technique is being used both as a research tool and to treat several non-motor disorders [7] and is becoming a promising tool for the treatment of anxiety [8], claustrophobia [9], fear of flying [10], arachnophobia [11] or post-traumatic stress symptoms [12], besides its use in more basic science to study the basis of decision making [13].

The relationship between VR and the human motor system is undoubtedly complex. However, it has been used to study the cognitive basis of complex movements such as navigation during locomotion [14], [15] and the interactions of execution/observation combined with fMRI [3]. Moreover, motor system pathologies can be evaluated through VR [16]. For example, the kinematics of hand movement in visual neglect patients [17], parkinsonian bradykinesia [18], or fractal analysis in physiological or pathological conditions [19], [20] have all been evaluated during VR immersion [21].

Several clinical studies have shown that arrhythmokinesis during finger tapping [22]–[24] is associated with un-steadiness of rhythm in complex movements like gait [25]. Arrhythmokinesis is characteristic of the elderly and of several pathologies like Parkinson's disease (PD) [24], and the typical alterations are present not only in the temporal but also in the spatial domain, such as movement amplitude. Interaction between visual and proprioceptive systems seems altered in pathological conditions; for instance, the integration of proprioceptive and visual information required to coordinate movements is impaired in PD [26]. In this sense, VR allows the researcher to control and modify the weight of the different sensory information resources to understand better the role of each in their current situation. VR is also considered to be a potential therapeutic tool in gait disorders after stroke [27] or phantom limb pain [28]; roles reinforced by evidence of neuroplasticity after VR therapy in cerebral palsy [29], [30] and pain relief [31].

With this background, we present an immersive HMD VR system by which the subject can be integrated in 1^st^ perspective (although 3^rd^ person perspective is also allowed), capturing their movements (finger taps) on-line, and placing them into the virtual world, aiming to shed some light on the following question: *Virtual reality and physical rehabilitation: a new toy or a new research and rehabilitation tool?* [32]. For this, it is of utmost importance to show that any VR system induces physiological responses in the same way that the real world does. This is a fundamental issue which needs to be dealt with prior to further presentation of more complex protocols to produce lasting physiological adaptations, or to use VR systems in experimental protocols including brain image evaluation [33], [34]. Since finger tapping in the real world is able to detect alterations in rhythm formation due to aging and PD [22]–[24], our aim is to understand if finger tapping in VR can reproduce such results. Our research hypothesis states that execution in a VR environment is reproducible across time and is capable of detecting alteration in movement features between the three different groups of subjects evaluated, PD patients, elderly healthy subjects, and young healthy subjects (as controls for the effect of aging).

## Materials and Methods

### Virtual Reality System

**Figure 1:**
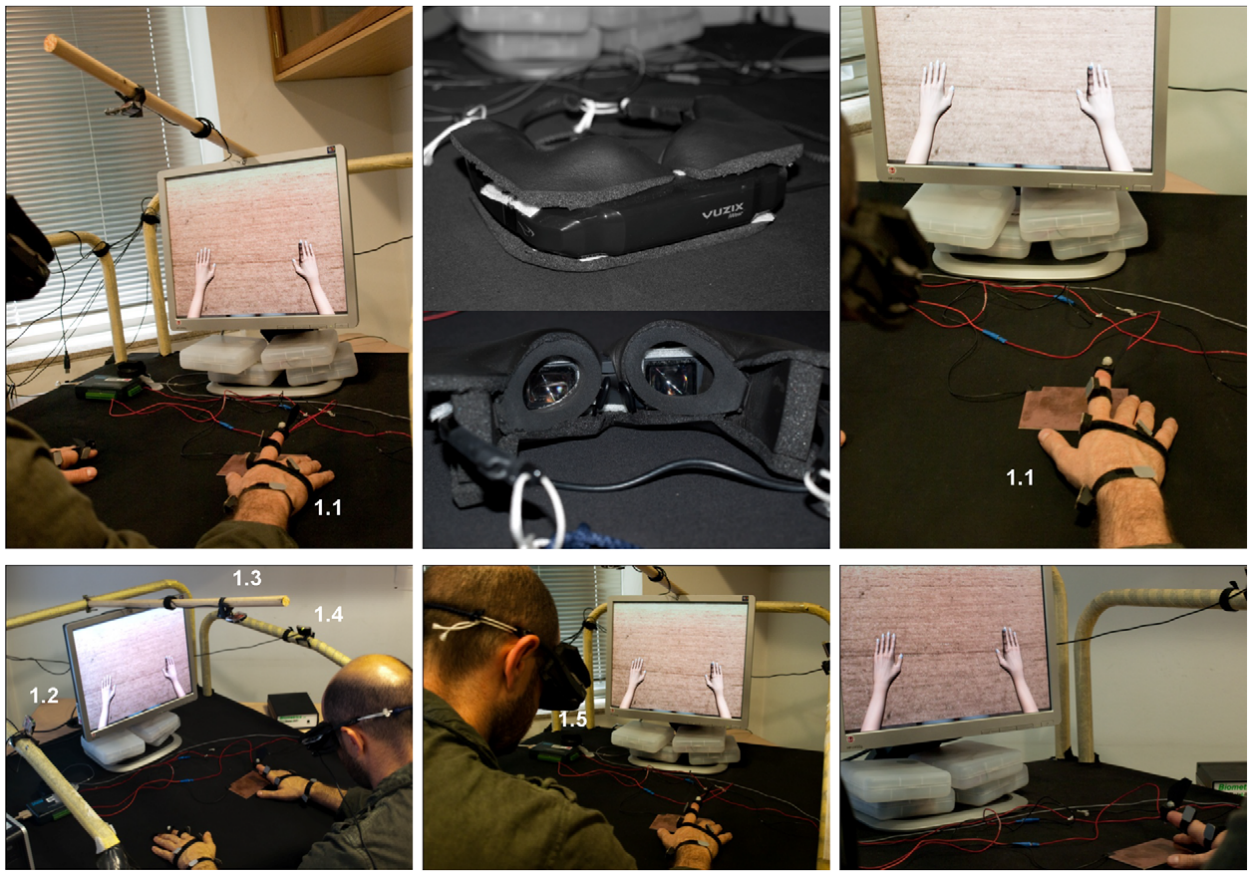
Virtual Reality System. Representation of the different elements in the system: movement of reflective markers at the Patient/Subject's anatomical points (1.1) are captured by IR cameras (1.2, 1.3, 1.4) and the cameras send information to the main computer, which integrates subjects movements into the virtual experience. An acquisition module (1.5) connects reciprocally to the main computer, so that different analogue and digital input/outputs can be controlled. The Subject wears the HMD, and the display is also shown on the main computer for experimenter to supervise the experience. The figure represents the virtual environment and the avatar's forearms in 1^st^ person perspective. The HMD is provided with tri-axial accelerometer tracking head movements and has been modified with black foam in order to isolate subjects completely from the real environment. The foam adapts to the subjects' face thereby “removing” the subject from the real world environment.

The subjects were seated on a chair, in a comfortable, relaxed posture, with their hands and forearms leaning on a table. The subject wore the HMD, which provided him with a first person view of a virtual environment which is similar to his actual environment: He sees a virtual room with a table in front of him and a virtual, generic depiction of himself (avatar) (Fig. 1). The frame may be zoomed in or out such that size of the avatar's hand can be adjusted to the real size of the subject's hand, enhancing immersion. The system tracks finger, hand and arm movements, and translates them realistically into the virtual character. To develop this realistic virtual environment, enhancing the sense of presence, we have chosen the Ogre3D engine (http://www.ogre3d.org), an open-source, scene-oriented, flexible 3D engine written in the C^++^ language. The main element in the 3D rendering program is the virtual avatar; a generic human-like 3D model was created using Maya and exported to Ogre format, with a significant level of detail ensuring the realistic look of the scene and preventing subjective disbelief.

The tracking system provides rotational and positional information from a series of markers placed on anatomical points (Fig. 1_1.1_). For tracking we have used 3 infra-red (IR) cameras, *TrackIR 4:PRO* from NaturalPoint, designed to track positional data from reflecting markers with a frequency of 120 frames/s, and a resolution of 320×288 pixels. The three cameras are specifically arranged to provide for a full three degree tracking for the X,Y,Z spatial positions of each tracking element (Fig. 1_1.2,1.3,1.4_).

The system directly registers the positional information returned by IR cameras, whereas rotational information is created from pairs of markers to obtain the value of each angle for the three axes. A special case-based interpolation algorithm based on statistic filtering was constructed in order to validate the stability of the system in situations where one of the hands may overlap. No markers are needed for the arms, since the specific spatial position of the hand is enough to interpolate rotation and position of arm, forearm or shoulder by using Inverse Kinematics techniques [35].

### Software architecture

Three separate modules were constructed for the system: The 3D rendering program, the control application and the camera control software. The former two are in the subject station, the latter on a second computer managed by the expert supervising the tests. To ensure proper communication among the three modules, a special communication system was also developed.

The supervision program uses a Graphical User Interface (GUI) based application which provides control over the whole system. Camera software, at the same time, communicates directly with the 3D program to supply the tracking information needed for motion capture. The 3D rendering software sends its state to the supervising program at all times so the expert can control the flow of the test. All three modules are connected through UDP non-blocking sockets using different application ports. The network can be laid out as a simple LAN Ethernet connection.

### Head Mounted Display

For a realistic, immersive experience, the subject wears the HMD. This is a pair of Vuzix iWear VR920 glasses which includes motion tracking, so that the virtual environment displayed is congruent with head movements (Fig. 1). Maximal resolution is 1024×768, which, along with AA filtering through GPU hardware, provides enough visual quality to ensure immersion. Tracking allows for yaw, pitch and roll of the head from an initial calibration stage. The HMD provides information by means of two screens, one for each of the eyes creating a stereoscopic 3-D vision. HMD Frame rate is 150 frames/s, exceeding the frame rate necessary to allow the visual system to perceive a continuous movement [36].

### Acquisition module

The system allows communication with any recording or stimulation external device. This is done by the *acquisition module* (Fig. 1 _1.5_, Advantech USB-4711A) which allows for 16 analog and digital inputs, and 8 analog and digital outputs. Information is gathered at real time thanks to the API provided.

### Experimental Design

Our goal was to examine if execution in the VR environment is a valid method to detect impairment in rhythm formation in the different groups (validity), and if the execution in the VR can be reproduced under the same conditions on different days (reliability). Further, consistency between VR and Real world data was assessed.

Subjects performed the same procedure twice, one week apart, under the same conditions. We included three different sets of subjects: Young healthy controls (YC), elderly healthy controls (EC), and Parkinson's disease (PD) patients. It has been shown that FT provides distinctive results in each of those groups [37].

### Subjects

#### Ethics Statement

All experimental subjects signed consent forms. The protocol conformed to the declaration of Helsinki and was approved by the Ethics Committee of the University of A Coruña (Spain) (CE-UDC 23/09-2009).

### Young Subjects

12 healthy subjects (based on medical history and personal interview; 7 men, 5 women) (mean age 24.3 yrs; SD: 4.9; range 18–35) were recruited from staff and students of our institution.

### Elderly Controls

12 EC (5 men, 7 women; mean age 66.6 yrs; SD: 10.1; range 51–85) healthy subjects (based on medical history and personal interview) were recruited from relatives of staff working in our institution.

### Parkinson's disease patients

10 (5 men, 5 women; mean age 69.9 yrs; SD: 11.2; range 51–89) idiopathic PD patients [38] were recruited. Participants belonged to the Parkinson's Disease Association in Bueu (Spain).

All participants were screened for dementia using MMSE [39] and Edinburgh Handedness Inventory (EHI) [40]. Subjects were excluded if they scored <24 in the MMSE or if they had any musculoskeletal impairment or disease apart from PD which might interfere with their ability to undertake the task.

PD patients were evaluated during OFF-periods (at least 12 h since their last anti-parkinsonian medication intake) and in the case of subjects taking controlled-released drugs the dose immediately before evaluation was withheld.

### Materials

Besides the VR system, n event detector, consisting of a conductive plate and a flexible conductive ring attached to the subject's distal phalanx, allowed recording of the tapping cycle, the calculation of the duration of the cycle (tapping frequency), and its Coefficient of Variation (CV). Information was sampled at 1 kHz.

### Procedure

Subjects performed the FT test in two different conditions: FT in the real environment (FT_REAL_) and FT in the VR environment (FT_VR_). FT_REAL_ and FT_VR_ were performed at each subjects preferred (*comfort*) tapping rate.

During the FT test subjects were comfortably seated with forearms pronated on a table in front of them, so that both elbows were flexed at about 90–100°. Seat height was adapted so that subjects were in an optimal comfort position to perform the test. Subjects were asked to perform FT with their index finger by flexing-extending the metacarpo-phalangeal joint while staring at the hand executing the task.

The same position was adopted by the avatar in the VR condition in the 1^st^ person perspective (egocentric perspective) [15], [41]. This allowed the avatar's forearms to be perceived as if they were the subjects own forearms (Fig. 1). Before starting the VR protocol the VR environment was zoomed in and out until each subject judged the avatar's hand size to be their own hand size.

Subjects performed the task with their dominant hand. In the case of PD patients all questions belonging to EHI were related to the period before the onset of symptoms, and they also performed with their dominant hand regardless the laterality of their signs. Subjects were instructed to look at the executing hand, whether in the virtual or real environment. For VR, correcting lenses (Bobes, Inc, Madrid) were attached to each of the two screens of the HMD if necessary. Three randomized sets of 50 cycles of FT_REAL_ and FT_VR_ (each) were performed, with resting periods of 3 minutes. None of the subjects reported fatigue. To allow subjects to reach a steady finger rate 3 taps were performed prior to acquiring the 50 to be analyzed.

Data for each variable, condition and day was obtained from the mean of the three repetitions performed during each session; work by Wu et al., evaluating intra-session variability during tapping in PD recommended a minimum of two sets [42].

### Variables analyzed

The variables analyzed were tapping frequency and Coefficient of Variation (CV) of inter-tap interval. The tapping frequency was calculated from the tapping events (expressed in Hz), and the CV of inter-tap interval was defined as follows:
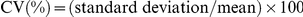



### Statistical Analysis

The statistical analysis included both tests of validity and reliability.

#### Validity

To evaluate the validity of FT_VR_ for detecting differences in rhythm formation between groups an ANOVA with repeated measures was used. Factor Group (between subjects factor) included three levels (each of the groups, PD, EC, YC). Testing was performed on two different days, so a factor DAY with two levels was included (DAY1 and DAY2).

#### Reliability

The reliability test compared the results obtained in VR on the first day of evaluation (D1) with those obtained on a second day (D2), one week later and under the same conditions; this was done for each variable and for each group.

We analyzed consistency in the execution in VR between the two days by means of Intra-class Correlation Coefficient (ICC), for each variable and group independently. The mean difference between days was also analyzed, (Day 1 minus Day 2 for each subject). This was evaluated by means of a one sample t-test and was evaluated for each group separately.

Further, we decided to compare consistency in execution in the VR vs. the Real world. This was done because Real world testing is the gold-standard in clinical practice. ICC and the mean difference between conditions (Real and VR world) were obtained for each group and variable independently and for each condition the average performance of the two days was used. Normality of distributions was assessed by means of one sample Kolmogorov-Smirnov test; if violated, a log transformation was applied. When using ANOVA, a univariate approach was used to analyze within subject effects, for this the Greenhouse-Geisser coefficient was used in order to correct the degrees of freedom in case of sphericity violation. Significance was set to 0.05. When subsequent pair-wise comparisons after ANOVA were performed, the Bonferroni correction was applied. Results were presented as Mean and Standard Deviation (SD).

## Results

### Virtual Reality System Validity and Reliability

#### Validity

**Figure 2:**
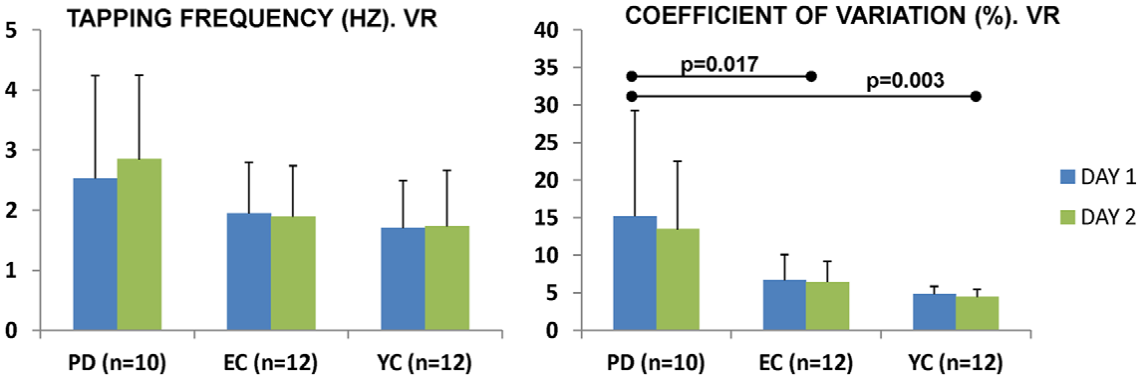
Motor behavior in the different days during VR testing. The figure represents the motor behavior on the first and second days of evaluation (blue and green respectively) for the different variables. Pair-wise comparisons after Bonferroni correction show that PD had larger variability that EC and YC on both days.

Execution in the VR was valid to detect differences in rhythm formation between groups. For the CV, Factor GROUP showed a significantly different ability to maintain tapping rhythm F(2,31) = 7.468 p = 0.002; Bonferroni correction for subsequent pair-wise comparisons showed larger variability in the PD vs. EC (p = 0.017), and PD vs. YC (p = 0.003); despite the EC showed larger variability that the YC the difference was not significant (p = 1.000). Factor DAY and DAY X GROUP interaction were not significant; this reflects that the groups' different ability to maintain a tapping rhythm was observed in both days (Fig. 2). Specifically, CV for the PD group was 15.20% (±14.10) on the first day, and 13.46% (±9.05) on the second. CV's for the EC group were smaller, 6.69% (±3.43) on the first and 6.44% (±2.74) on the second day. Finally, the smallest of the CVs were those of the YC, at 4.82% (±1.07) and 4.46% (±1.01) on days one and two, respectively.

Factor GROUP showed tapping frequencies were not significantly different between groups F(2,31) = 2.408 p = 0.107. Again, Factor DAY and the DAY X GROUP interaction were not significant (Fig. 2). PD patients tapped at an average of 2.53 Hz (±1.71) on the first day and at 2.85 Hz (±1.40) on the second; EC tapped at 1.95 Hz (±0.85) and at 1.89 Hz (±0.85) on the first and seconds days respectively; and the YC at 1.70 Hz (±0.79) and at 1.73 Hz (±0.93).

#### Reliability

**Figure 3:**
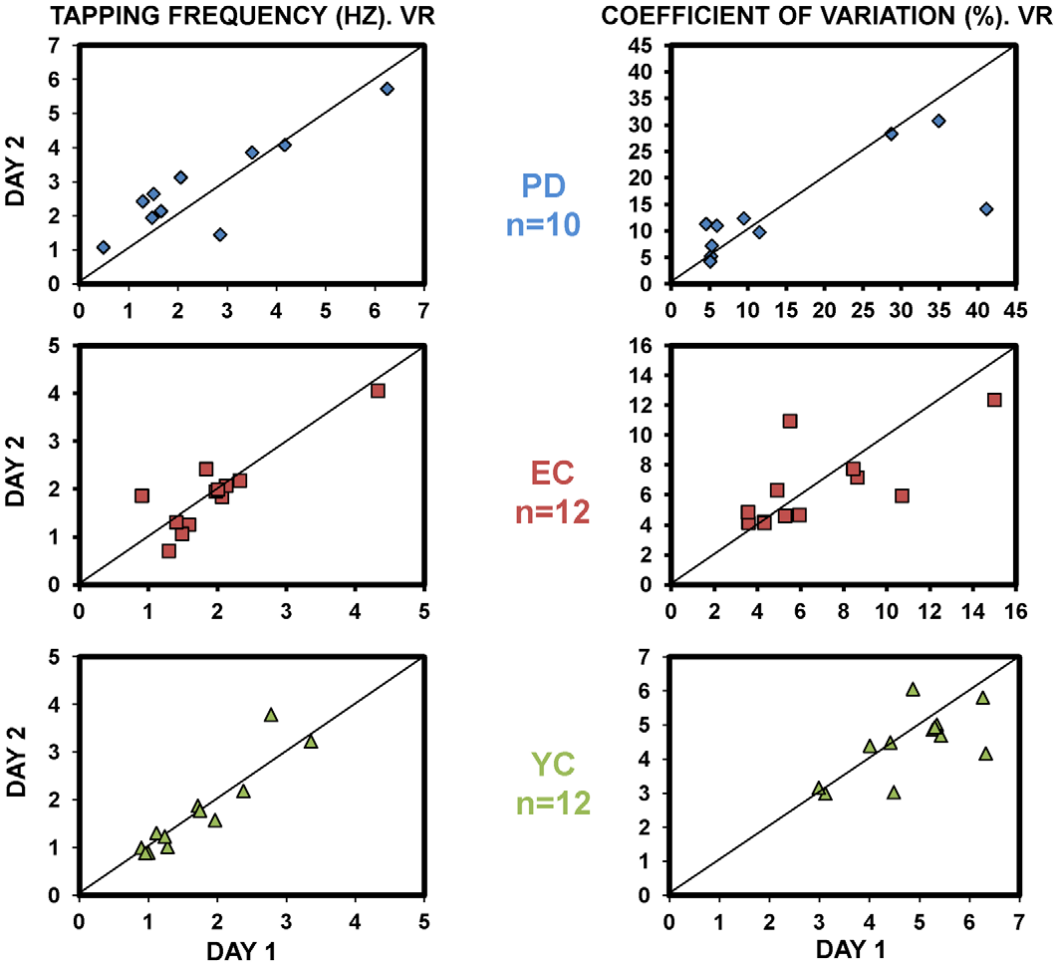
Scatter plot comparing execution between days in the VR environment. The figure illustrates the motor behavior on the first and second days of evaluation for each group and for both variables (Tapping Frequency left panel, CV right panel). ICC values between days, and 95% CI for the mean difference between days for each group and variable, are presented in tables.

Consistency in execution in the VR between day 1 and day 2 was evaluated by ICC and its 95% Confidence Interval. For CV, ICC was 0.87 with CI of [0.74; 0.93]; for the Tapping Frequency, ICC was 0.94 and CI [0.88; 0.97] (Fig. 3). ICC for the Frequency and CV for each group are shown in Table 1. The mean differences between days (MD_D1–D2_) were not significantly different from 0; this was observed for the CV, MD_D1–D2_ = 0.73% [−1.09; 2.54], and also for the Tapping Frequency, MD_D1–D2_ = −0.09 Hz [−0.27; 0.11] (Table 2).

**Table 1:** ICC between days during VR testing in the different groups.

	ICC D1 vs D2 [95% CI]			
	PD (n = 10)	EC (n = 12)	YC (n = 12)	GROUPS POOLED
TAPPING FREQUENCY (Hz)	0.92 [0.70; 0.98]	0.93 [0.77; 0.98]	0.96 [0.85; 0.99]	0.94 [0.88; 0.97]
CV INTERTAP INTERVAL (%)	0.81 [0.23; 0.95]	0.81 [0.35; 0.95]	0.80 [0.29; 0.94]	0.87 [0.74; 0.93]

**Table 2:** Mean difference between days for both the tapping frequency and the CV during VR testing.

	MEAN DIFFERENCE (D1–D2) AND [95%CI]			
	PD (n = 10)	EC (n = 12)	YC (n = 12)	GROUPS POOLED
TAPPING FREQUENCY (Hz)	−0.32 [−0.90; 0.25]	0.05 [−0.22; 0.33]	0.03 [−0.25; 0.20]	−0.09 [−0.27; 0.11]
CV INTERTAP INTERVAL (%)	1.73 [−5.02; 8.49]	0.26 [−1.31; 1.83]	0.35 [−0.19; 0.90]	0.73 [−1.09; 2.54]

**Figure 4:**
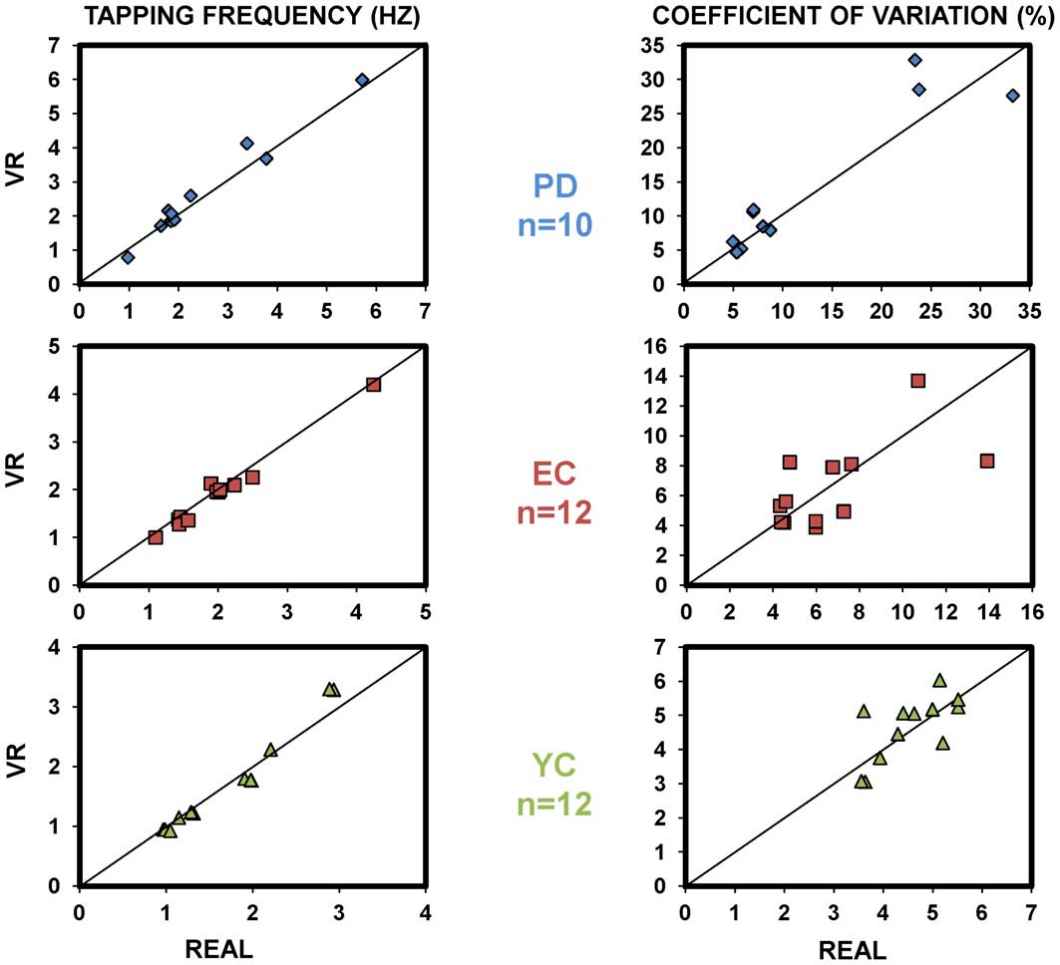
Scatter plot comparing execution in Real and VR environments. The figure shows the behavior in both conditions for each group and for both variables (Tapping Frequency left panel, CV right panel). ICC values and 95% CI for the mean difference between conditions (VR and Real), for each group and variable, are presented in tables.

Finally, we evaluated the consistency between execution in the Real world and VR, taking the average of day1 and day2 for each condition. This was done by means of ICC and its [95% CI]. It showed excellent results for the CV ICC = 0.96 [0.92; 0.98]; and for the Tapping Frequency ICC = 0.98 [0.97; 0.99] (Fig. 4; Table 3). The similarity between results obtained in the VR and the gold-standard reference Real world is reinforced by the fact that the mean differences between conditions were not significantly different from 0, this was observed either for the CV, MD_REAL-VR_ = −0.44% [−1.39; 0.51], and also for the Tapping Frequency MD_REAL-VR_ = −0.02 Hz [−0.10; 0.05] (Table 4).

**Table 3:** ICC between Real and VR testing in the different groups.

	ICC REAL vs. VR [95% CI]			
	PD (n = 10)	EC (n = 12)	YC (n = 12)	GROUPS POOLED
TAPPING FREQUENCY (Hz)	0.99 [0.96; 0.99]	0.99 [0.98; 0.99]	0.98 [0.94; 0.99]	0.98 [0.97; 0.99]
CV INTERTAP INTERVAL (%)	0.96 [0.84; 0.99]	0.77 [0.21; 0.94]	0.80 [0.30; 0.94]	0.96 [0.92; 0.98]

**Table 4:** Mean difference Real and VR for both the tapping frequency and the CV.

	MEAN DIFFERENCE (REAL-VR) AND [95%CI]			
	PD (n = 10)	EC (n = 12)	YC (n = 12)	GROUPS POOLED
TAPPING FREQUENCY (Hz)	−0.17 [−0.36; 0.02]	0.07 [−0.01; 0.15]	0.01 [−0.12; 0.13]	−0.02 [−0.10; 0.05]
CV INTERTAP INTERVAL (%)	−1.58 [−4.50; 1.34]	0.18 [−1.40; 1.76]	−0.11 [−0.55; 0.34]	−0.44 [−1.39; 0.51]

## Discussion

This study presents a VR system aimed at evaluating repetitive finger movements in healthy individuals and in subjects with neurological disturbances. A main outcome we have obtained here is that the system is reliable. This is important, since a basic requirement of any VR system to be useful in motor system research and potentially treat patients, is to provide stable responses under the same conditions. Further, evaluation in the VR environment was shown to be able to detect differences in behavior between groups. This is also relevant given that the task used is a basic test in human motor control, with clinical importance for the characterization of different pathological rhythmic patterns [37], [43]–[48]. In these conditions, FT in the VR mode can be used in complex research protocols to isolate subjects from non-natural environments (such as fMRI or PET scans; or complex neurophysiological laboratory settings). Finally, one basic criterion to be fulfilled in VR intervention is the sense of “presence” [7], [49], [50]. This was supported in our work by the fact the performance in the VR is consistent with that in the real world. Behavior of all three groups in VR displayed the same usual features reported in Real world. PD were prone to hasten during tapping [22], [24], and the characteristic differences in rhythm formation between groups [23] were revealed on both days during VR testing.

Although the system has allowed us to analyze the characteristics of a motor act, our ultimate goal is to modify the performance of a movement, especially in cases where movement is already pathologically altered. This may introduce motor adaptations, based on several sessions of VR, which might improve the typical motor disturbances observed in the elderly and in PD (e.g. arrhythmokinesis or hipometry). This is why the system permits modification of parameters of the movement performed by the avatar (amplitude, speed, frequency, etc..) which, hopefully, will improve motor impairments after imitation training [51], [52]. This hypothesis, which is ready be confirmed in the near future, seems to be supported by the excellent level of immersion observed (some subjects referred to the avatar's hand as “their own” hand as also by the fact that results in the real and the VR were identical and reproducible.

This system complements other approaches using VR for the study of different movements [52], [53], such as Haptic devices [54]. It matches the virtually recreated environment with the somatosensory information perceived by the subjects. When the avatar taps on the table the sense of touching is real since the subject is actually performing the task in synchrony. The sense of presence is also enhanced by scaling the size of the avatar's hand to match in size that of the experimental subject. Presence is reinforced by other features of the visual environment such as perspective or shades, and the high degree of isolation from the real world. The system is provided with a pair of glasses to which a foam-adapting edge was attached so that stray light and any other distractors from the real world are completely removed.

Here we have focused on a well known deterioration of the motor control due to aging and disease, the impairment in rhythm formation. As a marker we have used finger tapping at the preferred rate since tapping as fast as possible might increase variability due to fatigue [55], not reflecting impairment in rhythm formation. We acknowledge that this is a simple task (although with high value from the clinical point of view), and we also recognize that our work will be considered exploratory in nature and, in future, the use of larger sample sizes should confirm the results from this novel pilot study. Also, the inclusion of other variables (e.g. neurophysiological measurements) will increase the strength of our work and reinforce the utility of VR both to study and to treat motor disorders.

In conclusion, systems based on VR seem useful to study motor behavior. Specifically, the system presented here allows the evaluation of alteration in rhythm formation in PD and in the elderly. VR is sensitive in order to characterize different movement patterns. This allows inclusion of more complex virtual elements to interact either with the physiological and damaged motor system and its use in non-naturalistic environments such as brain image scans.
